# Molecular characterization of Peste des petits ruminants viruses in the Marmara Region of Turkey

**DOI:** 10.1111/tbed.13095

**Published:** 2018-12-28

**Authors:** Eda Altan, Satya Parida, Mana Mahapatra, Nuri Turan, Huseyin Yilmaz

**Affiliations:** ^1^ Veterinary Faculty Department of Virology University of Istanbul Istanbul Turkey; ^2^ Blood Systems Research Institute San Francisco California; ^3^ Department of Laboratory Medicine University of California at San Francisco San Francisco California; ^4^ The Pirbright Institute Pirbright, Woking, Surrey UK

**Keywords:** cattle, phylogenetic analysis, PPR, PPR in the Marmara region of Turkey and Bulgaria, real‐time RT‐PCR, sero‐prevalence, sheep

## Abstract

Recent outbreaks of Peste des petits ruminants (PPR) in the Marmara region of Turkey including the European part of Thrace is important due to its proximity to Europe (Greece and Bulgaria) and the potential threat of spread of PPR into mainland Europe. In order to investigate the circulation of PPRV in the region suspect clinical and necropsy samples were collected from domestic sheep (*n* = 211) in the Marmara region of Turkey between 2011 and 2012. PPR virus (PPRV) genome was detected in 10.4% (22 out of 211) of sheep samples by real‐time RT‐PCR, and PPR virus was isolated from lungs of two sheep that died from infection. Of the 22 positive samples nine were used for partial N‐gene amplification and sequencing. The phylogenetic analyses indicated that the virus belongs to lineage IV, the same lineage that is circulating in eastern and central part of Turkey since its first official report in 1999. In addition, samples from 100 cattle were collected to investigate potential subclinical circulation of PPRV. However all were found to be negative by real‐time RT‐PCR, and also in serological tests indicating the large ruminants were likely not exposed or infected with the virus. The impact of these findings on the potential threat of spread of PPR to Europe including the first PPR outbreak in Europe in Bulgaria on 23rd June 2018 is discussed.

## 1 INTRODUCTION

Peste des petits ruminants (PPR) is a highly contagious, acute, notifiable and economically important viral disease of small ruminants (goats and sheep). Following the successful eradication of rinderpest virus, the World Organization of Animal Health (OIE) and the Food and Agriculture Organisation (FAO) aim to globally eradicate the disease by 2030. PPR was long considered to be confined to West Africa but later it was described throughout Africa (with the exception of some southern African countries) from south of Sahara to north of Equator, as well as the Middle East and Asia (Banyard et al., 2010; Parida et al., 2015).

The causative agent PPR virus (PPRV) is an enveloped, non‐segmented negative sense single‐stranded RNA virus which is classified in the *Morbillivirus* genus of the *Paramyxoviridae* family (Gibbs, Taylor, Lawman, & Bryant, 1979). The viral genome consists of eight transcriptional units identified as 3′‐N‐P/C/V‐M‐F‐H‐L‐5′, and each gene is separated by intergenic regions. PPRV has a single serotype but it is genetically divided into four distinct lineages (I, II, III and IV) on the basis of partial sequence analysis of the fusion (F) protein gene (Forsyth & Barrett, 1995) and the nucleoprotein (N) gene (Couacy‐Hymann et al., 2002) favouring the N‐gene sequence comparison to F gene sequence comparison (Kumar et al., 2014).

**Figure 1:**
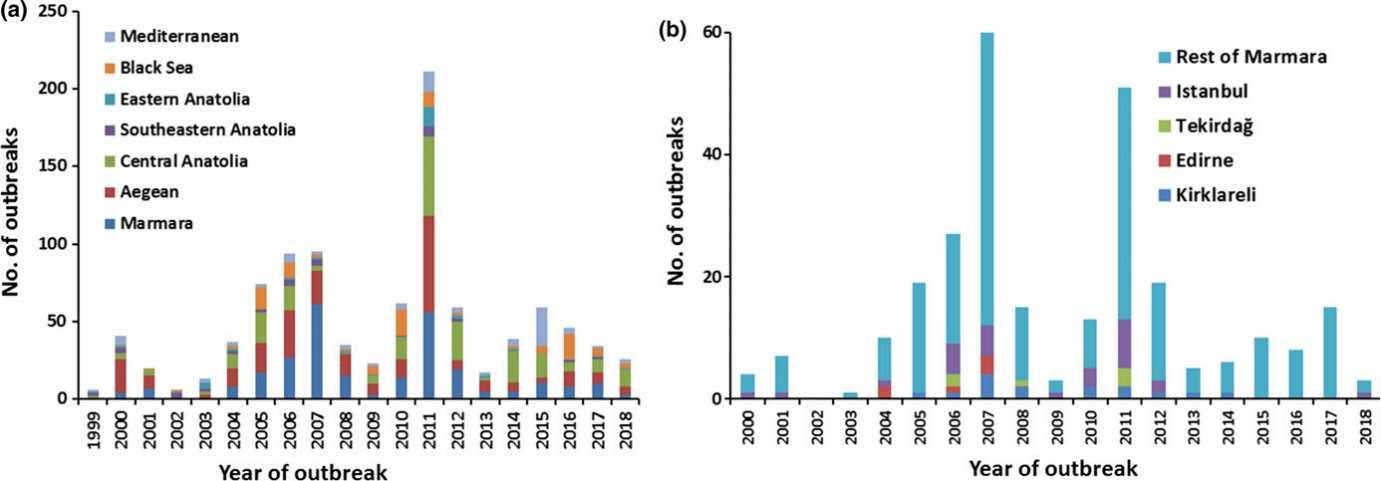
(a) PPR outbreaks reported in different regions of Turkey from 1999 to 2018 (2018: January to June). All data were taken from OIE official web site (http://www.oie.int/wahis_2/public/wahid.php/Diseaseinformation/statusdetail). (b) PPR outbreaks reported in different provinces in the Thrace region and rest of Marmara of Turkey from 2000 to 2018 (2018: January to June). All data were taken from OIE official web site (http://www.oie.int/wahis_2/public/wahid.php/Diseaseinformation/statusdetail) [Colour figure can be viewed at wileyonlinelibrary.com]

Turkey is located in the eastern Mediterranean and is a bridge between the continents of Europe and Asia. Turkey is among the highest small ruminant farming countries in the world, with an estimated sheep population of 31.5 million heads and a goat population of 10.4 million heads in 2016 (http://www.fao.org/faostat/en/?#data/QA retrieved on 24th of October, 2018). Europe is free of PPR. While this paper was at revision stage the first PPR outbreak was reported in Bulgaria on 23rd June 2018. In Turkey PPRV infection was first officially reported in southern and eastern Anatolia in 1999 (OIE, 1999). OIE has reported approximately 1,000 (997) PPR outbreaks in Turkey from 1999 to 2018 (Figure 1a). These outbreaks reached climax in 2007 and 2011 especially in the Marmara and Aegean regions of Turkey. In Thrace (the European part of Turkey), PPRV infection was reported in Istanbul in 2000 followed by Edirne (bordering Greece) in 2004 and Kırklareli (bordering Bulgaria) in 2006 (Figure 1b). The last outbreak was reported in 2014 on the European border in the Thrace region, and a total of 55 outbreaks were reported in this region since the first report of the disease (http://www.oie.int/wahis_2/public/wahid.php/Diseaseinformation/statusdetail). While this paper was at revision stage a new outbreak was reported in Istanbul (Figure 1b) in May, 2018. Similar to the situation in North Africa, outbreaks of PPR in Turkey pose a significant threat to Europe for the incursion of the disease (Parida et al., 2016). Therefore this study was designed to investigate the circulation of PPRV in domestic small ruminants (sheep) in the Marmara region of Turkey. In addition attempts were also made to look into the large ruminant population in the same region which may provide a snapshot of virus infection within populations where mild disease is present or where small ruminants are regularly vaccinated (Abubakar et al., 2017).

## 2 MATERIALS AND METHODS

### 2.1 Ethics statement

This study was approved by the Animal Experiments Ethics Committee of Istanbul University, Istanbul, Turkey and performed in strict accordance with the recommendations of the Animal Experiments Ethics Committee.

### 2.2 Study area and sample collection

**Figure 2:**
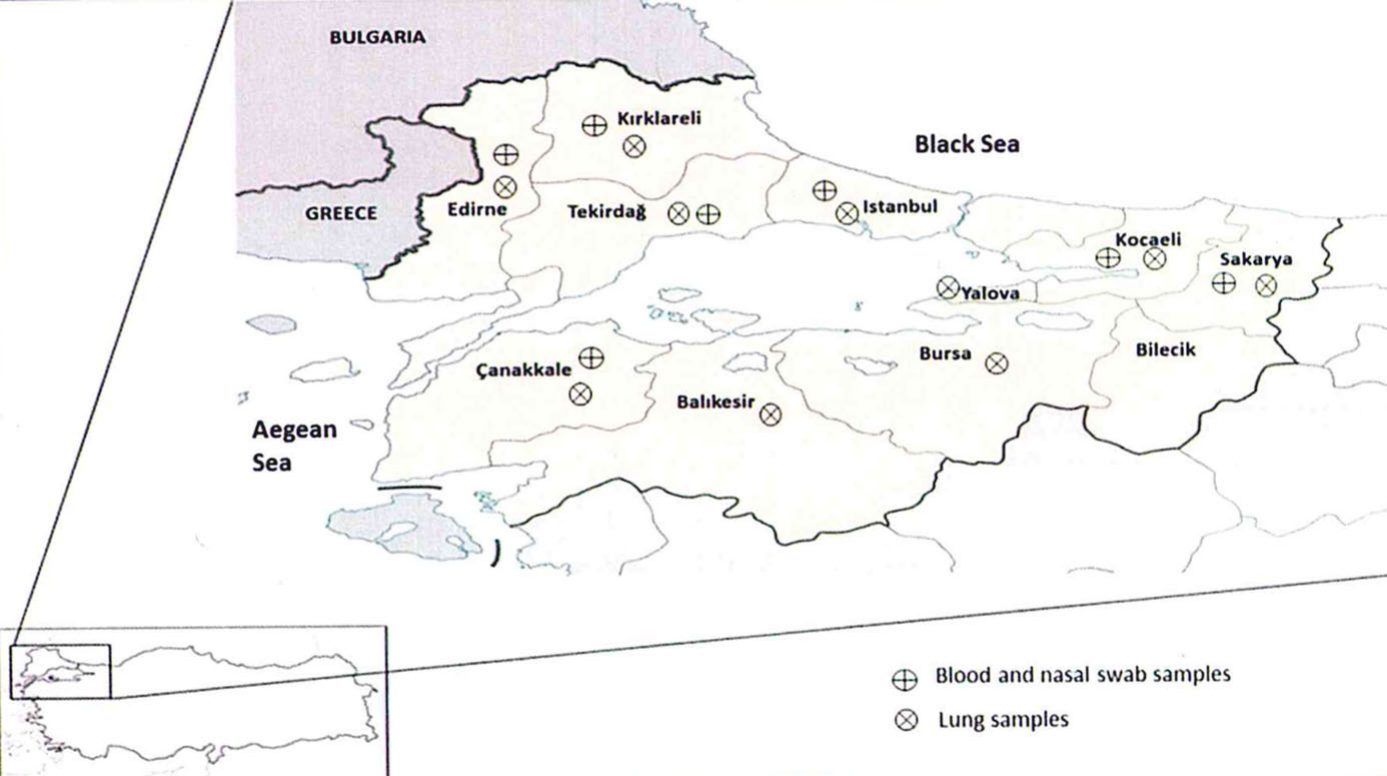
Districts of Marmara region of Turkey from where samples were collected in this study [Colour figure can be viewed at wileyonlinelibrary.com]

The Marmara region of Turkey bordering Europe was selected for this study. Samples were collected from sheep from 10 administrative districts (out of 11, except Bilecik province) in the Marmara region of Turkey, between June 2011 and March 2012 (Figure 2). A two stage sampling design was followed in which the first stage was taken forward through questionnaires to select the farms to sample, and the second stage was to select the animals to sample within each farm. The farms mainly contained sheep with a flock size of 50–300 animals; no goats were encountered in these farms during the course of the study. The animals were over 6 months of age and reported to be unvaccinated by the farmer. Within the districts, some farms practiced mixed farming involving the housing and maintenance of large and small ruminants in close contact. Following this design, a total of 19 farms from seven administrative districts, namely Canakkale (*n* = 2), Edirne (*n* = 1), Istanbul (*n* = 6), Kirklareli (*n* = 1), Kocaeli (*n* = 2), Sakarya (*n* = 1) and Tekirdag (*n* = 6) were selected and biological samples were collected from a total of 111 animals (both male and female) selected randomly from each farm taking blood and nasal swabs from each animal. From each farm, a maximum number of 15 animals were sampled depending on the flock size. Within the district where PPR like symptoms (fever, respiratory distress, ocular and nasal discharge, weakness, diarrhoea etc.) were observed in some farms, attempts were made to collect samples from other farms where animals were not showing any PPR like symptoms with the exception of Edirne and Kirklareli. In addition, lung samples were collected from 100 sheep (about 2–12 months old) from 10 administrative districts at post‐mortem that died with respiratory distress symptoms or slaughtered for meat purpose (Table 1) making it a total of 211 samples.

**Table 1:** Details of the samples and results of biological samples **(**nasal swab, blood and lung tissue) collected from sheep for this study

Provinces in the Marmara region	Nasal swab and blood samples							Lung tissues		
	No. of blood and nasal swabs tested	No. of positive sera	No. of positive, only blood sample	No. of positive, only nasal swab	No. of positive in both blood and nasal swab	No. of blood samples sequenced	No. of nasal swabs sequenced	No. of lung samples tested	No. of positive	No. of samples sequenced
Edirne^a^	10 (10)	−	−	−	−	NT	NT	7 (1)	−	NT
Kirklareli^a^	3 (3)	−	−	−	−	NT	NT	5 (4)	2	−
Tekirdağ^a^	25 (8)	−	−	−	−	NT	NT	17 (10)	3	−
Istanbul^a^	35 (28)	4	−	2	1	−	2	23 (16)	4	4
Çanakkale	21 (15)	7	1	−	2	1	−	11 (6)	−	NT
Bursa	−	−	−	−	−	NT	NT	2 (2)	−	NT
Balıkesir	−	−	−	−	−	NT	NT	7 (7)	1	−
Yalova	−	−	−	−	−	NT	NT	4	−	NT
Kocaeli	10 (2)	2	1	2	−	1	−	16 (9)	−	NT
Sakarya	7	−	−	−	−	NT	NT	8 (3)	3	1
Total	111 (66)	13	2	4	3	2	2	100 (58)	13	5

−: negative; NT: not tested.

a Indicates provinces in the Thrace region of Turkey. The numbers in parenthesis indicates the samples collected from sheep showing PPR like clinical symptoms (fever, respiratory distress, purulent occular and nasal discharges, weakness, diarrhoea)/pathological lesions (pneumonia, haemorrhage in lung) wherever applicable.

Cattle and buffalo can also be infected with PPRV without showing any clinical symptoms and sero‐convert (Ozkul et al., 2002; Abraham et al., 2005; Abubakar et al., 2017). Following the same sampling strategy blood and nasal swabs were collected from 50 cattle from 13 farms from seven administrative districts, namely Canakkale (*n* = 2), Edirne (*n* = 1), Istanbul (*n* = 4), Kirklareli (*n* = 1), Kocaeli (*n* = 2), Sakarya (*n* = 1) and Tekirdag (*n* = 2) to account for subclinical infection. The farms had a flock size of 30–200 animals and were different to the farms where samples from sheep were collected. In each farm, a maximum number of 6 animals were sampled depending on the flock size. In addition, lung samples from 50 slaughtered cattle originating from the same region were also collected making it a total of 100 samples (Table 1).

Samples were transported to the laboratory on ice (4–8°C). The swabs and tissues were stored at −70°C until used while blood samples were processed immediately. The buffy coats from the blood samples with EDTA were collected and stored at −70°C for RNA extraction. Serum was separated from clotted blood and stored at −20°C until tested.

### 2.3 Antibody detection

Sera collected from sheep (*n* = 111) and cattle (*n* = 50) were tested for the presence of PPRV‐specific antibodies using the anti‐nucleoprotein (N) PPRV competitive ELISA (cN‐ELISA, ID Screen^®^ PPR Competition ELISA, ID.Vet, France). The assays were performed and analysed following the manufacturer's instructions. Samples with percentage inhibition (PI) value <50% were considered positive. All the tests were carried out in duplicate, and the borderline positive samples were repeat tested to confirm results. The average of two results was used in subsequent analysis.

### 2.4 Virus isolation

For isolation of PPR virus from lung tissues the tissues were processed following the method as previously described (Clarke, Mahapatra, Friedgut, Bumbarov, & Parida, 2017). The filtered lung homogenate was used for RNA extraction and also for virus isolation on Vero.DogSLAM (VDS) cells. The cells were checked every day for the appearance of PPRV‐specific cytopathic effect (CPE) for 7 days, and at least 4 blind passages were carried out before the samples were declared negative.

### 2.5 RNA extraction and real‐time reverse transcription‐Polymerase chain reaction (qRT‐PCR)

All clinical samples were screened for the presence of viral nucleic acid by qRT‐PCR. For the extraction of total RNA from cell culture supernatant, buffy coat and nasal swabs, QIAamp Viral RNA mini kit (Qiagen) was used whereas RNeasy mini kit (Qiagen) was used for tissue samples. Extraction procedures were carried out according to the instructions of the manufacturer. The presence of PPRV nucleic acids in these samples was determined by qRT‐PCR following the method of Kwiatek et al. (2010) using Superscript III Platinum R one step qRT‐PCR system kit (Invitrogen).

### 2.6 Reverse Transcription (RT), Polymerase Chain Reaction (PCR) and nucleotide sequencing

Samples found positive by qRT‐PCR were selected for PCR and sequencing. The viral RNA was reverse transcribed and the C‐terminus of the N‐gene was amplified as previously described (Baazizi et al., 2017). The PCR amplicons were purified using the QIAEXII PCR purification kit (Qiagen) according to the manufacturer's instructions and sent to a commercial company (REFGEN, Ankara, Turkey) for sequencing. Sequences were assembled and analysed using SeqMan II (DNAStar Lasergene 8.0). Alignments of the N‐gene sequences were made using the Clustal W program and used for construction of distance matrices using the Kimura 2‐parameter nucleotide substitution model (Kimura, 1980) as implemented in the programme MEGA 6.0 (Tamura, Stecher, Peterson, Filipski, & Kumar, 2013). A maximum‐likelihood phylogenetic tree was then generated using MEGA 6.0, and the robustness of tree topology was assessed using 1,000 bootstrap replicates.

### 2.7 Data analysis

The data analysis was carried out using Minitab 7.0 software.

## 3 RESULTS AND DISCUSSION

In Turkey PPRV infection was first officially reported from Southeastern Anatolia, Eastern Anatolia and Mediterranean region in October 1999, and from the Aegean region in December of the same year (OIE, 1999) though there had been some previous reports of the presence of the virus in the country (Alcigir, Atalay Vural, & Toplu, 1996; Tatar, 1998; Ozkul et al., 2002). The following year (2000) PPRV infection was reported from all of the regions in Turkey including Istanbul (part of Thrace in the Marmara region). The outbreaks spread to other provinces in Thrace, Edirne in 2004 and Kirklareli in 2006. In the year 2006, two further outbreaks were recorded in Tekirdag province of Thrace. Since then PPRV outbreaks have occurred in different regions of Turkey including Marmara every year although the last outbreak in Thrace region was recorded in 2014 in Kırklareli (Figure 1b)**.** Though the Thrace region was free of PPR for 3 years (2015–2017), the disease reappeared in the Thrace region of Turkey again; one PPR outbreak was reported in Istanbul in May 2018 (http://www.oie.int/wahis_2/public/wahid.php/Diseaseinformation/statusdetail).

The presence of PPR in Turkey is significant because of the unique geographical position of the country and the threat to bordering countries where large population of naïve animals exist (Parida et al., 2016). Following the continuous outbreaks in Turkey since 1999 the General Directorate of Food and Control division of Turkey's Ministry of Food, Agriculture and Livestock was engaged in a European Union funded project titled “Tagging and Vaccination of Sheep and Goats (project no: TR 0802.08)”. The project continued for 3 years starting in spring of 2010 and aimed at (a) identification/registration of the entire sheep and goat populations nationwide; and (b) control of PPR by means of efficient vaccination policies. During this project (a) approximately 65 million small ruminants (both existing and new populations) were ear tagged and registered in a database in order to be able to trace their movements in line with the EU requirements; (b) 90 million small ruminants were vaccinated against PPR over 3 years. Although the project did not succeed in eliminating PPR from the region, it significantly reduced the PPR outbreaks in Turkey including the Marmara region.

Among the 111 sheep sampled for nasal swabs and blood in this study, 66 sheep (59.45%) exhibited PPR like clinical symptoms such as respiratory distress, purulent ocular and nasal discharges, weakness and diarrhoea. Similarly amongst 100 sheep sampled for lung tissue at post‐mortem, pneumonia and haemorrhages in the lungs were observed in 48 (48%) sheep.

Out of 111 sheep sera analysed in this study, antibodies to PPRV were detected in 11.7% sheep. These sera originated from 4 different farms in the Marmara region, one from Kocaeli, two from Istanbul and one from Canakkale (Table 1), but none from the provinces (Edirne, Kirklareli and Tekirdag) immediately bordering Greece and Bulgaria. This indicates prior circulation of PPRV in the region as these animals have not been previously vaccinated. This is not surprising as PPR outbreaks have been reported continuously in the Thrace region of Turkey from 2006 to 2014 (Figure 1b). Both low and high PPR antibody sero‐prevalence (2.75%–74%) have been reported in many countries by using ELISA (Al‐Naeem, Elzein, & Al‐Afaleq, 2000; Singh, Saravanan, Sreenivasa, Singh, & Singh, 2004; Zahur et al., 2008; Balamurugan et al., 2011). The percent of seropositive animals found in this study is lower than that (14.9% and 87.9%) previously reported in Turkey (Tatar & Alkan, 1999; Ozkul et al., 2002; Tatar et al., 2002; Albayrak & Alkan, 2009). Comparison of antibody sero‐prevalence results of this study and previous studies in Turkey shows that the prevalence of PPR in the Marmara region, especially in Thrace is much less in comparison to other areas in Turkey.

All the samples collected in this study were subjected to qRT‐PCR in order to detect presence of PPRV nucleic acid. Of the 211 sheep samples tested in this study 22 (10.4%) were found to be positive with threshold cycles values (*C*_*T*_) values ranging from 22 to 38. Of these nine (9) samples (blood: 2, nasal swabs: 4 and both blood and nasal swabs: 3) were biological samples collected from live animals whereas the remainder 13 were from the lung tissue samples (out of 100 samples) collected during necropsy (Table 1). All the biological samples (*n* = 38) collected from the provinces (Edirne, Kirklareli and Tekirdag) immediately bordering Greece and Bulgaria were found to be negative in qRT‐PCR; however five lung samples (out of 29 samples), two from Kirklareli and three from Tekirdag, were positive albeit with very high *C*_*T*_ values (above 35). All the samples positive in qRT‐PCR (*n* = 22) were subjected to conventional RT‐PCR to amplify a 351 bp product of C‐terminus of the N gene. A total of nine amplified products were obtained that originated from five lung tissues, two nasal swabs and two blood samples. The *C*_*T*_ value of the samples that were amplified in conventional PCR was between 22 and 32 while the remainder that could not be amplified ranged from 33 to 38.

A total of nine (9) partial N‐gene sequences were generated in this study and submitted to NCBI (GenBank accession number KJ764831, KJ764832, KJ764833, KJ797011, KJ797012, KJ797013, KJ797014, KJ797015, KJ797016). All the partial N‐gene sequences (*n* = 7) from the lungs and blood samples were identical. However, the two samples from the nasal swabs had two nucleotide differences (synonymous changes) compared to the sequences from the lungs and blood samples resulting in 99.2%–100% identity at nucleotide level among the viral sequences generated in this study. Further, all the viral sequences (available in NCBI database) from the Marmara region (*n* = 26) collected between 2010 and 2011 were analysed, and found to be 98.8%–100% identical at nucleotide level. The partial N‐gene sequences generated in this study were further compared to the sequences of the historic strains from Turkey (Turkey 96 and Turkey 2000). Compared to the first virus characterized from Turkey available in NCBI (Turkey 96 ‐ DQ840184) the sequences from samples isolated in 2011 (this study) were 4% different at nucleotide level whereas they were 2.4% different to Turkey 2000 (AJ563705) sequence.

**Figure 3:**
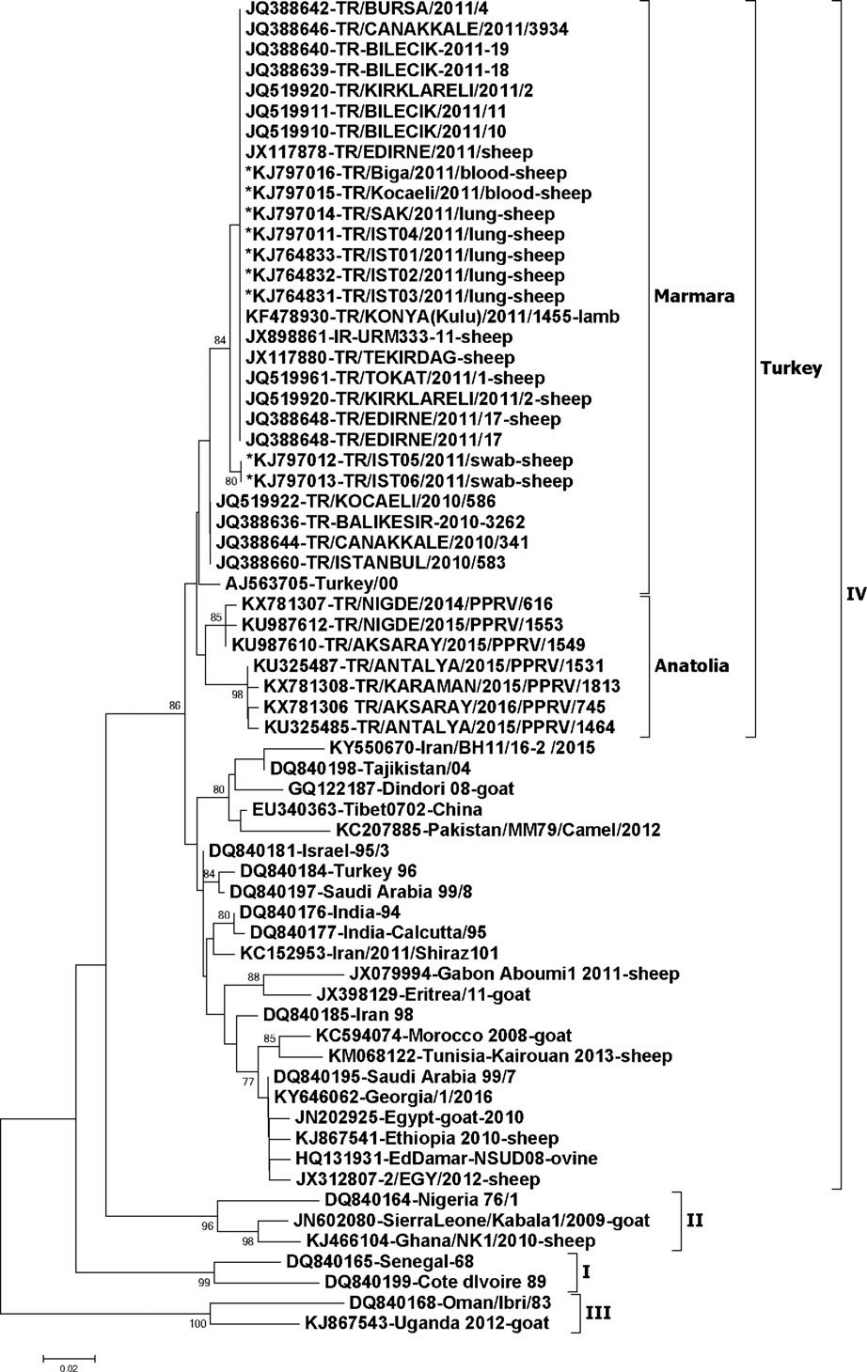
Neighbor‐joining tree constructed on the basis of partial N‐gene sequences of peste des petits ruminants virus (PPRV), showing relationships among the PPRV isolates from Turkey. The Kimura 2‐parameter model was used to calculate percentages (indicated by numbers beside branches) of replicate trees in which the associated taxa clustered together in 1,000 bootstrap replicates. The sequences generated in this study are indicated by an asterisk at the begining of the taxon name. Scale bar indicates nucleotide substitutions per site

The partial N‐gene sequences of the neighbouring countries available in NCBI database were retrieved to be included in the phylogenetic analysis. The sequences that contained the full 255 nucleotides at similar position of the N‐gene (*n* = 56) were only selected making it a total of 65 sequences. A phylogenetic analysis of the partial N‐gene sequences (Figure 3) indicated that all the sequences generated in this study were of lineage IV, as described previously (Ozkul et al., 2002; Yesilbag, Yilmaz, Golcu, & Ozkul, 2005; Guler, Sevik, & Hasoksuz, 2014; Sevik & Sait, 2015) and were in the same sub‐cluster of Turkey‐2000. However, previously reported Turkey 1996 strain clustered together with viral sequences from Israel, India and Saudi Arabia. Recently, 13 viral sequences originating between years 2014–2016 from central Anatolia and Antalya province of Turkey were made available in GenBank, and retrieved to be included in the analysis. The identical sequences were excluded resulting in addition of only seven sequences in the analysis. These sequences clustered within the Turkey cluster, but separate to the sub‐cluster of Marmara viruses. These 2014–2016 viruses were 2.8%–3.98% different at nucleotide level when compared to the 2011 Marmara PPR viral sequences generated in this study.

Live infectious virus was isolated from two lung samples on 3rd day of inoculation using VDS cells and on 5th day using Vero cells (data not shown). Culture supernatants from those cells were tested in qRT‐PCR and were found to be strongly positive.

No clinical symptoms were observed in cattle sampled in this study. During necropsy at the slaughter house no PPRV‐specific pathological findings were observed. In serological tests no antibodies to PPRV were detected in cattle sera. All the samples were also negative in qRT‐PCR assays. This indicates that the large ruminants have not been infected with the PPR virus meaning there is no continuous circulation of PPR virus in cattle in the region. In comparison to PPR virus circulation in cattle in other endemic countries i.e. Pakistan (10%; Abubakar et al., 2017) and India (11%; Balamurugan et al., 2014) it is reassuring that the Marmara region of Turkey is not yet intensely endemic for PPR. The occasional outbreaks of PPR in the Thrace region could be due to the movement of infected sheep and goats from other endemic regions of Turkey.

While this paper was at revision stage a PPR outbreak was reported in Bulgaria (1st PPR outbreak in the EU) on 23rd June 2018 in sheep in Bolyarovo municipality in Jambol region on the border with the Thrace region of Turkey (Supporting information Figure S1). In a flock of 540 sheep (*n* = 380) and goats (*n* = 160), 2 sheep tested positive and one died, with disease confirmed by national reference laboratory, Bulgaria and later on by the EU reference laboratory, CIRAD, France. Control and eradication measures included stamping out of the infected herds, including preventive culling of all other small ruminants reared in the village (about 800 sheep and goats, in total), the establishment of protection and surveillance zones, movement restriction and intensified surveillance in the municipalities located along the border of the country with 3rd countries not free from PPR (http://oie.int/wahis_2/public/wahid.php/Diseaseinformation/WI). The second outbreak of PPR was confirmed in Kosti village in Burgas region on 28th June, 2018 (Supporting Information Figure S1). Stamping out policy included culling of all small ruminants reared in the village. Ten kilometre zones were established and enhanced surveillance was implemented (http://oie.int/wahis_2/public/wahid.php/Diseaseinformation/WI). Further, four new outbreaks of PPR were reported in Krainovo (*n* = 1), Strandzha (*n* = 1) and Sharkovo (*n* = 2) in Bolyarovo on 9th July, 2018 in addition to the first two outbreaks. As with earlier outbreaks, stamping out and preventive culling were carried out (http://oie.int/wahis_2/public/wahid.php/Diseaseinformation/WI). The distance between Turkish border and Bolyarovo, Bulgaria is about 20 km. Therefore it is possible that the source of the outbreak could be from Turkey because of either illegal movement of sheep and goats incubating the disease due to free border between the countries including transport of animals by less obvious route such as a boat returning from Turkey to Bulgaria or the spread of the disease through wildlife (Parida et al., 2016). The viruses currently causing PPR outbreaks in Bulgaria have not been defined yet; however ongoing molecular epidemiological studies in reference laboratories, i.e. sequencing the causative agent, could confirm the origin of the virus.

In conclusion, PPR is endemic in Turkey including the Marmara region. There is a potential risk of spread of PPR to Europe as a large number of small ruminants are transferred within Turkey, particularly from areas of high endemicity to the Marmara region of Turkey. In addition there is another potential route of transmission as Turkey is directly connected to mainland Europe, and hosts the same community of wild ruminants (susceptible to PPR virus) as Europe including, roe deer and red deer. These animals are widespread and abundant wild ruminants in Europe, including Bulgaria and Greece bordering Turkey, and may serve as a bridging species for PPRV transmission between distant populations of infected and healthy domestic sheep and goat populations (Parida et al., 2016). There is also evidence of wild goats and white‐tailed deer being either clinically or experimentally infected by PPRV (Hoffmann et al., 2012; Hamdy & Dardiri, 1976). Further from our unpublished data we have recorded wild boars and warthogs in Africa are positive for PPR antibodies. Recently, suids including wild boar have been reported to be experimentally clinically infected with PPRV (Schulz, Fast, Schlottau, Hoffmann, & Beer, 2018). However, at the moment the role of wildlife in the spread of the disease is unknown, and may warrant a survey in future. Therefore continuous monitoring of the disease, tracing of movement of animals in the Marmara region and maintaining a buffer zone in Thrace by regular vaccination is essential to prevent spread of the disease to Europe.

## CONFLICT OF INTEREST

Authors declare that they have no conflict of interest.

## STATEMENT OF ANIMAL RIGHTS

There was no ethical issue concerning this study. Animals were humanly treated during sampling.

## INFORMED CONSENT

Authors were informed and consent was approved.

## References

[tbed13095-bib-0001] Abraham, G. , Sintayehu, A. , Libeau, G. , Albina, E. , Roger, F. , Laekemariam, Y. , … Awoke, K. M. (2005). Antibody seroprevalences against peste des petits ruminants (PPR) virus in camels, cattle, goats and sheep in Ethiopia. Preventive Veterinary Medicine, 70, 51–57. 10.1016/j.prevetmed.2005.02.011 15967242

[tbed13095-bib-0002] Abubakar, M. , Mahapatra, M. , Muniraju, M. , Arshed, M. J. , Khan, E. H. , Banyard, A. C. , … Parida, S. (2017). Serological detection of antibodies to Peste des Petits ruminants virus in large ruminants. Transboundary and Emerging Diseases, 64, 513–519. 10.1111/tbed.12392 26200233 PMC5347956

[tbed13095-bib-0003] Albayrak, H. , & Alkan, F. (2009). PPR virus infection on sheep in blacksea region of Turkey: Epidemiology and diagnosis by RT‐PCR and virus isolation. Veterinary Research Communications, 33, 241–249. 10.1007/s11259-008-9172-5 18787968

[tbed13095-bib-0004] Alcigir, G. , Atalay Vural, S. , & Toplu, N. (1996). First pathological and immunohistological description of pest of small ruminants virus infection in lambs in Turkey. Veteriner Fakultesi Dergisi, Ankara Universitesi, 43, 181–189.

[tbed13095-bib-0005] Al‐Naeem, A. , Elzein, E. M. E. A. , & Al‐Afaleq, A. I. (2000). Epizootiological aspects of peste des petits ruminants and rinderpest in sheep and goats in Saudi Arabia. Revue Scientifique Et Technique De L Office International Des Epizooties, 19, 855–858. 10.20506/rst.19.3.1261 11107629

[tbed13095-bib-0006] Baazizi, R. , Mahapatra, M. , Clarke, B. D. , Ait‐Oudhia, K. , Khelef, D. , & Parida, S. (2017). Peste des petits ruminants (PPR): A neglected tropical disease in Maghreb region of North Africa and its threat to Europe. PLoS ONE, 12, e0175461 10.1371/journal.pone.0175461 28426782 PMC5398521

[tbed13095-bib-0007] Balamurugan, V. , Krishnamoorthy, P. , Raju, D. S. N. , Rajak, K. K. , Bhanuprakash, V. , Pandey, A. B. , … Rahman, H. (2014). Prevalence of Peste‐des‐petits‐ruminant virus antibodies in cattle, buffaloes, sheep and goats in India. Virusdisease, 25, 85–90. 10.1007/s13337-013-0177-5 24426314 PMC3889243

[tbed13095-bib-0008] Balamurugan, V. , Saravanan, P. , Sen, A. , Rajak, K. K. , Bhanuprakash, V. , Krishnamoorthy, P. , & Singh, R. K. (2011). Sero‐epidemiological study of peste des petits ruminants in sheep and goats in India between 2003 and 2009. Revue Scientifique Et Technique‐Office International Des Epizooties, 30, 889–896. 10.20506/rst.30.3.2087 22435199

[tbed13095-bib-0009] Banyard, A. C. , Parida, S. , Batten, C. , Oura, C. , Kwiatek, O. , & Libeau, G. (2010). Global distribution of peste des petits ruminants virus and prospects for improved diagnosis and control. Journal of General Virology, 91, 2885–2897. 10.1099/vir.0.025841-0 20844089

[tbed13095-bib-0010] Clarke, B. , Mahapatra, M. , Friedgut, O. , Bumbarov, V. , & Parida, S. (2017). Persistence of Lineage IV Peste‐des‐petits ruminants virus within Israel since 1993: An evolutionary perspective. PLoS ONE, 12, e0177028 10.1371/journal.pone.0177028 28545149 PMC5436660

[tbed13095-bib-0011] Couacy‐Hymann, E. , Roger, F. , Hurard, C. , Guillou, J. P. , Libeau, G. , & Diallo, A. (2002). Rapid and sensitive detection of peste des petits ruminants virus by a polymerase chain reaction assay. Journal of virological methods, 100, 17–25. 10.1016/S0166-0934(01)00386-X 11742649

[tbed13095-bib-0012] Forsyth, M. A. , & Barrett, T. (1995). Evaluation of polymerase chain reaction for the detection and characterisation of rinderpest and peste des petits ruminants viruses for epidemiological studies. Virus Research, 39, 151–163. 10.1016/0168-1702(95)00076-3 8837881

[tbed13095-bib-0013] Gibbs, E. P. , Taylor, W. P. , Lawman, M. J. , & Bryant, J. (1979). Classification of peste des petits ruminants virus as the fourth member of the genus Morbillivirus. Intervirology, 11, 268–274. 10.1159/000149044 457363

[tbed13095-bib-0014] Guler, L. , Sevik, M. , & Hasoksuz, M. (2014). Phylogenetic analysis of peste des petits ruminants virus from outbreaks in Turkey during 2008‐2012. Turkish Journal of Biology, 38, 671–678. 10.3906/biy-1401-28

[tbed13095-bib-0015] Hamdy, F. M. , & Dardiri, A. H. (1976). Response of white‐tailed deer to infection with peste des petits ruminants virus. Journal of wildlife diseases, 12, 516–522. 10.7589/0090-3558-12.4.516 16502689

[tbed13095-bib-0016] Hoffmann, B. , Wiesner, H. , Maltzan, J. , Mustefa, R. , Eschbaumer, M. , Arif, F. A. , & Beer, M. (2012). Fatalities in wild goats in kurdistan associated with peste des petits ruminants virus. Transboundary and Emerging Diseases, 59, 173–176. 10.1111/j.1865-1682.2011.01270.x 22074184

[tbed13095-bib-0017] Kimura, M. (1980). A simple method for estimating evolutionary rates of base substitutions through comparative studies of nucleotide sequences. Journal of molecular evolution, 16, 111–120. 10.1007/BF01731581 7463489

[tbed13095-bib-0018] Kumar, K. S. , Babu, A. , Sundarapandian, G. , Roy, P. , Thangavelu, A. , Kumar, K. S. , … Parida, S. (2014). Molecular characterisation of lineage IV peste des petits ruminants virus using multi gene sequence data. Veterinary microbiology, 174, 39–49. 10.1016/j.vetmic.2014.08.031 25248690

[tbed13095-bib-0019] Kwiatek, O. , Keita, D. , Gil, P. , Fernandez‐Pinero, J. , Clavero, M. A. , Albina, E. , & Libeau, G. (2010). Quantitative one‐step real‐time RT‐PCR for the fast detection of the four genotypes of PPRV. Journal of virological methods, 165, 168–177. 10.1016/j.jviromet.2010.01.014 20116402

[tbed13095-bib-0020] OIE (1999). Peste des petits ruminants in Turkey. Disease Information, 12, 137.

[tbed13095-bib-0021] Ozkul, A. , Akca, Y. , Alkan, F. , Barrett, T. , Karaoglu, T. , Dagalp, S. B. , … Burgu, I. (2002). Prevalence, distribution, and host range of Peste des petits ruminants virus, Turkey. Emerging Infectious Diseases, 8, 708–712. 10.3201/eid0807.010471 12095439 PMC2730320

[tbed13095-bib-0022] Parida, S. , Muniraju, M. , Altan, E. , Baazizi, R. , Raj, G. D. , & Mahapatra, M. (2016). Emergence of PPR and its threat to Europe. Small Ruminant Research, 142, 16–21. 10.1016/j.smallrumres.2016.02.018 27695194 PMC5035059

[tbed13095-bib-0023] Parida, S. , Muniraju, M. , Mahapatra, M. , Muthuchelvan, D. , Buczkowski, H. , & Banyard, A. C. (2015). Peste des petits ruminants. Veterinary microbiology, 181, 90–106. 10.1016/j.vetmic.2015.08.009 26443889 PMC4655833

[tbed13095-bib-0024] Schulz, C. , Fast, C. , Schlottau, K. , Hoffmann, B. , & Beer, M. (2018). Neglected hosts of small ruminant morbillivirus. Emerging infectious diseases, 24, 2334–2337. 10.3201/eid2412.180507 30457523 PMC6256395

[tbed13095-bib-0025] Sevik, M. , & Sait, A. (2015). Genetic characterization of peste des petits ruminants virus, Turkey, 2009‐2013. Research in Veterinary Science, 101, 187–195. 10.1016/j.rvsc.2015.05.005 26022069

[tbed13095-bib-0026] Singh, R. P. , Saravanan, P. , Sreenivasa, B. P. , Singh, R. K. , & Singh, B. (2004). Prevalence and distribution of peste des petits ruminants virus infection in small ruminants in India. Revue Scientifique Et Technique‐Office International Des Epizooties, 23, 807–819. 10.20506/rst.23.3.1522 15861876

[tbed13095-bib-0027] Tamura, K. , Stecher, G. , Peterson, D. , Filipski, A. , & Kumar, S. (2013). MEGA6: Molecular evolutionary genetics analysis version 6.0. Molecular biology and evolution, 30, 2725–2729. 10.1093/molbev/mst197 24132122 PMC3840312

[tbed13095-bib-0028] Tatar, N. (1998). Koyun ve kecilerde kucuk ruminatlarn vebasi ve sigir vebasi enefeksiyonlarinin serolojik olarak arastirilmasi. 1998. PhD thesis, Ankara University, Ankara, Turkey. Ankara University,Ankara, Turkey

[tbed13095-bib-0029] Tatar, N. , & Alkan, F. (1999). Serological and virological investigation of rinderpest and pest of small ruminants virus in sheep and goats. Etlik Veteriner Mikrobiyoloji Dergisi, 10, 35–60.

[tbed13095-bib-0030] Tatar, N. , Erturk, A. , Kabakli, O. , Akkoca, N. , Incoglu, S. , Ulker, U. , & Dakman, A. (2002). Investigation of peste des petits ruminants prevalence in Turkiye. Etlik Veteriner Mikrobiyoloji Dergisi, 13, 15–31.

[tbed13095-bib-0031] Yesilbag, K. , Yilmaz, Z. , Golcu, E. , & Ozkul, A. (2005). Peste des petits ruminants outbreak in western Turkey. Veterinary Record, 157, 260–261. 10.1136/vr.157.9.260 16127137

[tbed13095-bib-0032] Zahur, A. B. , Irshad, H. , Hussain, M. , Ullah, A. , Jahangir, M. , Khan, M. Q. , & Farooq, M. S. (2008). The epidemiology of peste des petits ruminants in Pakistan. Revue Scientifique Et Technique‐Office International Des Epizooties, 27, 877–884. 10.20506/rst.27.3.1848 19284055

