# Supplementary figures and images for: Molecular characterization of Peste des petits ruminants viruses in the Marmara Region of Turkey
Source: Transbound Emerg Dis. 2018 Dec 28;66(2):865–72. doi: 10.1111/tbed.13095 (PMC7814889; doi:10.1111/tbed.13095)

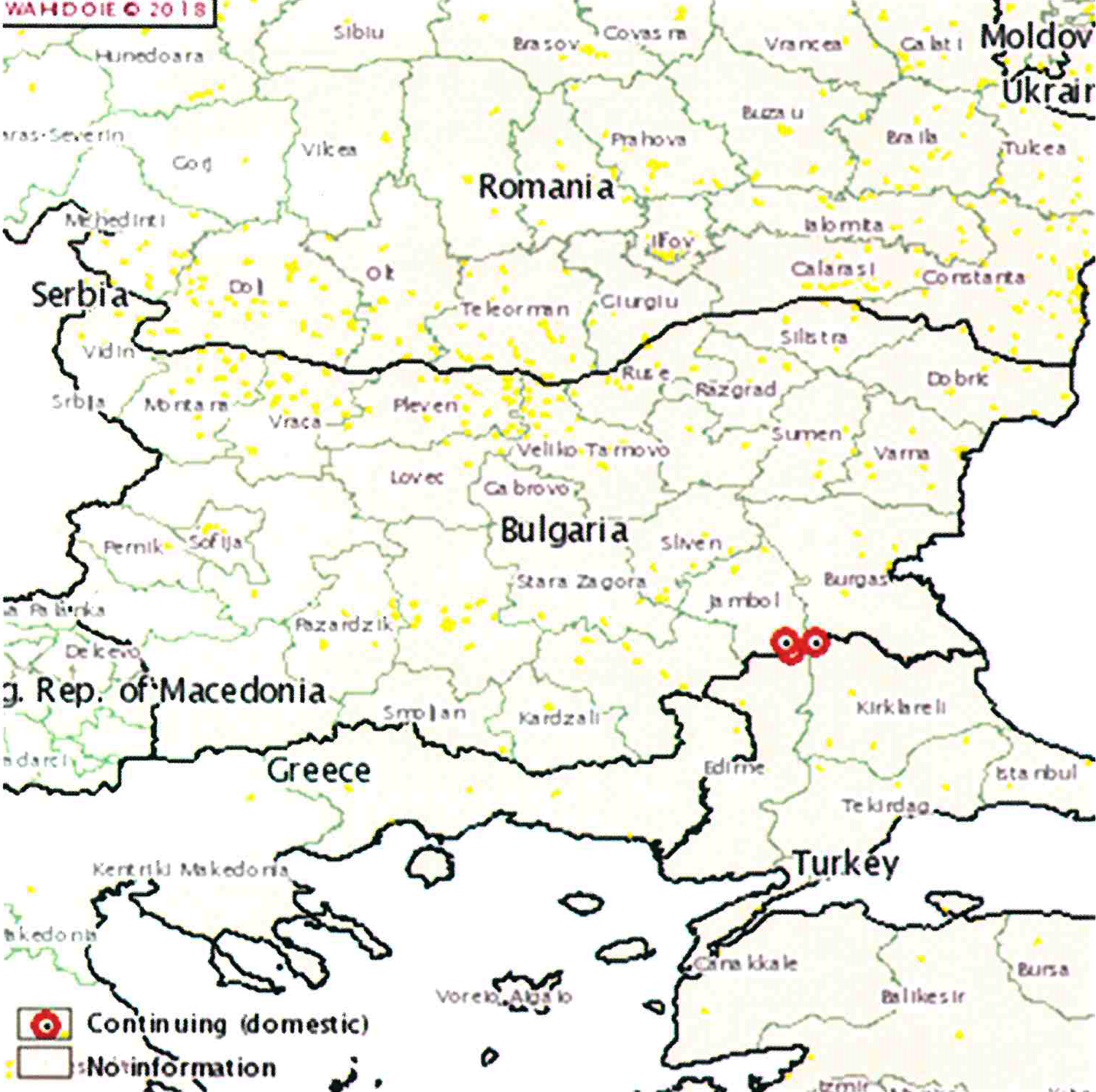

Supplement: Supplementary file 1 [file TBED-66-865-s001.tif]
